# Quantitative Analysis of Porcine Reproductive and Respiratory Syndrome (PRRS) Viremia Profiles from Experimental Infection: A Statistical Modelling Approach

**DOI:** 10.1371/journal.pone.0083567

**Published:** 2013-12-17

**Authors:** Zeenath U. Islam, Stephen C. Bishop, Nicholas J. Savill, Raymond R. R. Rowland, Joan K. Lunney, Benjamin Trible, Andrea B. Doeschl-Wilson

**Affiliations:** 1 The Roslin Institute & R(D)SVS, University of Edinburgh, Edinburgh, Midlothian, United Kingdom; 2 Institute of Immunology and Infection Research, University of Edinburgh, Edinburgh, United Kingdom; 3 Department of Diagnostic Medicine and Pathobiology, College of Veterinary Medicine, Kansas State University, Manhattan, Kansas, United States of America; 4 United State Department of Agriculture, Beltsville Agricultural Research Center, Beltsville, Maryland, United States of America; Virginia Polytechnic Institute and State University, United States of America

## Abstract

Porcine reproductive and respiratory syndrome (PRRS) is one of the most economically significant viral diseases facing the global swine industry. Viremia profiles of PRRS virus challenged pigs reflect the severity and progression of infection within the host and provide crucial information for subsequent control measures. In this study we analyse the largest longitudinal PRRS viremia dataset from an in-vivo experiment. The primary objective was to provide a suitable mathematical description of all viremia profiles with biologically meaningful parameters for quantitative analysis of profile characteristics. The Wood's function, a gamma-type function, and a biphasic extended Wood's function were fit to the individual profiles using Bayesian inference with a likelihood framework. Using maximum likelihood inference and numerous fit criteria, we established that the broad spectrum of viremia trends could be adequately represented by either uni- or biphasic Wood's functions. Three viremic categories emerged: cleared (uni-modal and below detection within 42 days post infection(dpi)), persistent (transient experimental persistence over 42 dpi) and rebound (biphasic within 42 dpi). The convenient biological interpretation of the model parameters estimates, allowed us not only to quantify inter-host variation, but also to establish common viremia curve characteristics and their predictability. Statistical analysis of the profile characteristics revealed that persistent profiles were distinguishable already within the first 21 dpi, whereas it is not possible to predict the onset of viremia rebound. Analysis of the neutralizing antibody(nAb) data indicated that there was a ubiquitous strong response to the homologous PRRSV challenge, but high variability in the range of cross-protection of the nAbs. Persistent pigs were found to have a significantly higher nAb cross-protectivity than pigs that either cleared viremia or experienced rebound within 42 dpi. Our study provides novel insights into the nature and degree of variation of hosts' responses to infection as well as new informative traits for subsequent genomic and modelling studies.

## Introduction

Porcine reproductive and respiratory syndrome (PRRS) is one of the most important infectious diseases threatening pig production worldwide [1]. PRRS reduces reproductive performance in breeding animals and increases respiratory problems in animals of all ages, leading to impaired growth in young piglets and, in some cases, mortality [2]–[4]. Infection with the PRRS virus (PRRSV) results in viremia and virus replication in multiple organs within the host; the targets for replication are macrophages in various tissues, primarily the lung but also in lymph nodes, spleen, placenta and umbilical cord [5]–[7]. One of the most significant challenges facing the eradication of the disease is the persistent nature of the etiological agent, PRRSV, which may persist within the host for several weeks or months, in some cases maintaining a sub-clinical lifetime persistence [8], [9]. If the persistently PRRSV infected individuals also remain infectious, they can drive the epidemiological dynamics of the disease within the population through perpetuating the cycle of transmission to susceptible animals [10].

Viremia profiles of *in-vivo* experimentally PRRSV challenged pigs are valuable indicators of the severity and progression of the infection in the host, and thus provide crucial information for the required subsequent disease control measures [11]. The course of a typical PRRSV infection is characterised by an acute viremic stage lasting approximately 4 weeks followed by a stage characterised by low levels and eventual resolution of viremia. Previous studies suggest that in the majority of animals viremia reaches undetectable levels typically by 4–6 weeks, although the virus may still be isolated months later in the lymphoid tissues [12], [13].

PRRSV challenge experiments with longitudinal viremia measures reveal substantial differences in the viremia profiles between hosts infected with the same PRRSV challenge dose, pointing to considerable variation in the host response to PRRSV infections. For example, numerous studies have shown breed differences in viremia levels and duration and also in antibody production [11], [14]–[16]. Reiner et al.[17] observed that Pietrain pigs infected with an attenuated PRRSV strain had longer viremia lasting until 72 days post infection (dpi), and a less efficient antibody production than Miniature pigs whose viremia only lasted up to 35 dpi. Viremia was classified as persistent in Pietrain pigs, however the profiles revealed both uni- and biphasic curves which could be a manifestation of viremia reactivation from the original infection within the host or reinfection between the pigs [18]. Using longitudinal viremia records collected over a 42 day period from 531 pigs challenged with a virulent PRRSV strain, Boddicker et al. [19]reported substantial differences between individual viremia profiles and also in total viremia, summarised as “area under the curve” (AUC) or viral load (VL). Furthermore, based on visual inspection, they classified pigs into two categories, i.e. non-rebounders and rebounders, characterised by mono- and bi-phasic serum viral profiles, respectively.

Given the apparent diversity in viremia patterns, several important questions arise. For example, for vaccine development or consideration of genetic disease control strategies it is important to determine whether and to what extent the observed differences in the profiles are influenced by the host and the virus genotype. In the longitudinal study of Boddicker et al. [20], the VL measure was found moderately heritable (h^2^ = 0.3), pointing to significant host genetic influence underlying disease severity and progression. Rebound was however not found to be heritable and thus thought to be controlled more by the virus than the host genotype [20]. However, the low heritability estimate of this trait (0.03) may have arisen due to the limited dataset, insufficient observations to capture the rebound phase, or the potential misclassification of individuals based on visual inspection of the profiles. Furthermore, there may be other profile characteristics representing host genetic variation in specific immune functions.

One hallmark of PRRSV is its high genetic diversity due to its fast mutation rate, resulting in continuous emergence of new quasi-species that may evade the host's immune system [21], [22]. Antibodies with PRRSV-neutralising activity usually appear from 14–28 days post infection (dpi) and are correlated with the reduction of PRRSV in the lung and the peripheral blood [23]. Despite conflicting reports on the significance of neutralizing antibodies (nAb) in anti-PRRSV protection [24], one would expect that diversity in the nAb response is important for cross-protective immunity against different PRRSV isolates, mutants or quasi-species. Thus, it would be useful to know whether host differences in viremia patterns are also reflected by differences in the breadth of nAb, and whether these measures are directly related. For example, one may hypothesize that a host that is able to clear the virus faster may have a less diverse nAb response than hosts with persistent or bi-phasic viremia profiles experiencing more cycles of virus replication and mutation.

From an epidemiological perspective, viremia rebound may constitute a problem as pigs diagnosed as cleared may have high levels of infectious virus a few days later. It would be useful to know whether viremia rebound reflects genuinely viremia reactivation rather than fluctuations in circulating virus load or measurement errors, and whether all or only a subset of pigs experience rebound. In particular, it would be useful to know whether virus rebound, or persistence, can be predicted based on early serum profile characteristics.

Addressing the questions raised above would require frequent repeated measurements of viremia, as well as of nAb diversity, on a large number of pigs subjected to the same experimental PRRSV challenge conditions. Such data are now available from the PRRS Host Genetic Consortium [1]. However, raw viremia data are inherently noisy and incomplete. Empirical mathematical functions have proven a useful tool for smoothing noisy data profiles and for exploring characteristics of dynamic patterns [25], [26]. Thus, an appropriate mathematical function may be able to concisely represent the full range of viremia profiles using only a few parameters. In particular, functions in which individual parameters represent specific curve characteristics provide an opportunity to apply rigorous statistical analysis to quantify differences in viremia patterns.

The primary objective of this study was to find mathematical functions that adequately represent the full range of viremia profiles obtained from a large scale PRRSV infection experiment, and use these to determine quantitative characteristics of infection dynamics. The functions will be used to derive an objective method of classification of viremia profiles based on statistical inference and to assess the relationship between the breadth of nAb response and viremia profile.

## Materials and Methods

### Experimental Data

The data analysed in this study was obtained from the PRRS Host Genetic Consortium (PHGC) trials, the largest PRRSV in-vivo challenge study to date; a detailed description of the experimental protocol is outlined in [1], [27]. Briefly, viremia data was obtained from pigs which were experimentally infected with NVSL 97–7985, a virulent isolate of PRRSV, [28], in eight separate infection trials (ca. 200 pigs/trial) with an infection dose of 10^5^ tissue culture dose_50_ (TCID_50_). The challenged pigs came from high health farms that were free of PRRSV, *Mycoplasma hypopneumoniae* and swine influenza virus. Pigs were placed randomly in pens of 10–15 pigs and were infected with PRRSV after a 7 day acclimation period, i.e. at 0 days post infection (0 dpi). Blood samples were collected immediately before infection (0 dpi) and at 4, 7, 11, 14, 19/21, 28, 35, 40/42 dpi and the level of PRRS viremia was measured using a semi-quantitative TaqMan PCR assay for PRRSV RNA. The viremia quantity data from RT-PCR was transformed on the logarithmic scale to the base 10 before the model fitting. Due to the sensitivity of RT-PCR the threshold of detection was set at 1 units on the log_10_ scale [20].

For the purpose of this study, only individuals with a minimum of 6 serum viremia observations were retained. This resulted in a viremia dataset comprising 1371 pigs in total, with over 170 pigs per trial, with the exception of trial 6 for which data from 89 pigs with less than 6 viremia observations had to be discarded. The majority of missing observations in Trial 6 were from 14 dpi onwards due to the outbreak of a bacterial infection, thus reducing the potential to capture viremia rebound. All individuals from Trials 7 and 8 were missing the last observation at 40/42 dpi due to management issues in the experimental facility.

To explore hypotheses surrounding the association between viremia profiles and the variability of the nAb response to PRRSV, nAB data from serum collected at 42 dpi was obtained for 490 individuals from the first three trials using a virus neutralization assay as outlined in [29]. Briefly, serum neutralising assays were conducted to examine the presence of cytopathic effects on the homologous PRRSV strain as used in the *in-vivo* challenge experiment (NVSL-7985 denoted henceforth as NVSL) and three additional PRRSV isolates: KS06-72109 (KS06), P-129 and VR-2332 (VR). These type 2 PRRSV isolates were chosen for genetic differences based on viral ORF5 sequence. KS06 had been isolated in 2006 and as a more contemporary isolate is still found in the field. Excluding the relatedness between P129 and NVSL (95%), nucleotide comparisons within ORF5 show that the PRRSV isolates differed from each other by 10% or greater. Each serum sample was reacted with the panel of four type 2 viral isolates, where the NVSL isolate served as the homologous virus in the serum neutralisation assays. Serum samples were considered positive for PRSSV nAb at a titre of eight or higher. Individuals in PHGC Trials 1–3 were assigned to one of the following five nAb categories: 1) failed to produce a nAb response, 2) only produced antibodies against the challenge virus (homologous nAb response), 3) produced nAb against the original and a different isolate (mild heterologous response), 4) produced nAb against the original and two different isolates (moderate heterologous response), 5) produced nAb against all four isolates (broad response). Analysis was conducted on the combined nAb categories of no cross protection (categories 1–2) and cross-protection (categories 3–5) as this was the most biologically relevant grouping of the nAb classes.

### Viremia Profile Characteristics and Mathematical Models

**Figure 1:**
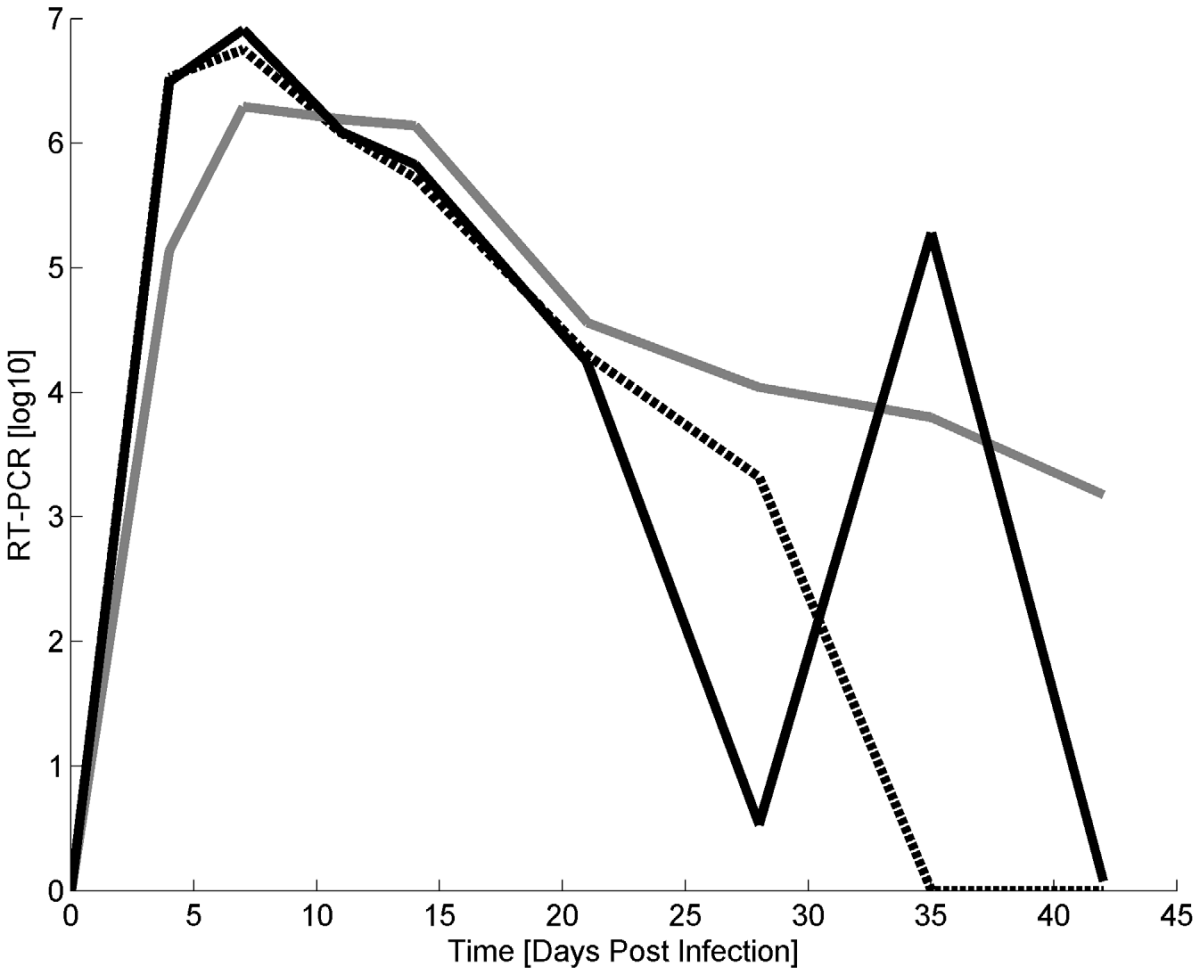
Raw phenotypic data profiles of three representative pigs experimentally infected with PRRSV. The viremia profile categories are: undetectable within 42 dpi (black dotted line), mono-phasic experimental persistence up to 42 dpi (grey line ), and bi-phasic rebound (black line).

Visual inspection of the individual viremia profiles from the raw data indicated that the profiles can be empirically grouped into three categories, illustrated in Figure (1): undetectable viremia levels at 42 dpi, persistence up to 42 dpi, and apparent clearance within the first 35 dpi followed by viremia rebound. Thus, observed profiles were either uni- or biphasic. Apparent viremia rebound occurred from 28 dpi onwards. A suitable mathematical model should thus be able to represent both uni-modal clearance and persistence as well as biphasic viremia rebound. Despite large inter-host variation in the individual profiles, visual inspection further indicated that all non-rebound viremia profiles and the primary phase of rebound profiles are characterised by a relatively rapid viremia increase towards the peak followed by a gradual exponential decline. Furthermore inter-host variation in the profiles is initially small, but increases over time.

### Wood's Model

The Wood's function, a gamma-type function often used to empirically describe lactation curves in dairy cattle [25], [30], [31], was chosen as candidate model as it appears to satisfy the above described data characteristics of the uni-modal profiles and the primary phase of the biphasic profiles. The function is given in equation (0.1): 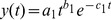
(0.1)where 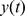 represents the level of viremia in the blood (log_10_ RT-PCR) at 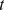 days post infection (dpi). The constant 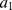 is a scalar quantity and impacts upon the magnitude of all the points on the curve. The parameter 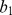 is an indicator of the initial rate of increase to the peak viremia level and the parameter 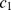 is an indicator of the rate of decline after the peak and dominates the function as 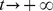. The maximum viremia load is achieved at time 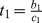 dpi, and the value of the maximum viremia is
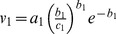
. Other curve characteristics, such as the rate of viremia decline at any point in time or the cumulative viremia load up to a given time t post infection can be readily obtained by differentiation or integration of the above function.

### Extended Wood's Model

In order to represent the bi-phasic profiles, the Wood's function was extended to a biphasic function described by equation (0.2):
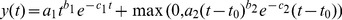
(0.2)where the model parameters 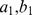 and 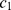 define the primary phase of the rebound profile as described for the Wood's model above. Time 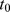 denotes the onset of the second phase of the profile, which is assumed to follow the same (Wood's) shape as the primary phase and is thus defined by the second set of Wood's model parameters: 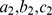. For 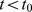 the Extended Wood's model is equivalent to the Wood's model. The Extended Wood's model has the derived parameters denoting the time and value of the first and second peaks respectively: 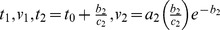. Similarly, rates of viremia decline or cumulative viremia load at any time post infection can be calculated through differentiation and numerical integration of the Extended Wood's function.

### Model Fitting and Parameter Estimation

Both the Wood's and Extended Wood's function were fitted to the individual data profiles using Bayesian inference with a likelihood framework. This was implemented by using an adaptive, population based Markov chain Monte Carlo method with power posteriors as described in [32]–[35]. The prior distributions for the function parameters were assumed to be uniformly distributed within a biologically realistic range. Parameters were estimated separately for each pig. The resulting inferences are based on 3000 samples, thinned from 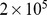 iterations of a non-adaptive Markov chain, with the first half of the chain discarded as burn in. For observations greater than the RT-PCR measurement threshold of 1 units on the log_10_ scale the errors were assumed normally distributed around 0 with a standard deviation of 0.5 log units, and for observations less than or equal to the RT-PCR observation threshold the errors were assumed cumulative normal.

Both the Wood's and Extended Wood's functions were fitted to all pigs. Thus the fitting procedure provided for each individual pig posterior distributions for every parameter of the Wood's and Extended Wood's function, respectively, from which parameter means, modes and credibility intervals were derived.

### Assessment of Model Fit

The accuracy of the model fit and choice of best model was assessed based on the Akaike's Information Criterion (AIC), together with inspection of the predicted model profiles with 95% posterior credibility intervals for every individual, to gain insight into both the accuracy and potential bias of the model predictions over the time course of the experiment. This included plotting histograms of the parameter estimates, inspection of the residuals, as well as calculating the (product moment) correlation between observations and predictions at each sampling time point.

### Objective Classification of Profiles

Due to the high degree of fluctuation in the viremia measurements, classification of the raw data profiles into the three categories shown in Figure 1 is somewhat arbitrary and not always straight-forward. However, the two alternative mathematical viremia models, i.e. the Wood's and the Extended Wood's model, together with statistical measures of goodness-of-fit provided an objective method for assigning the profiles into uni-modal and biphasic categories.

An individual was classified as experiencing viremia rebound if the biphasic Extended Wood's model had a statistically superior fit to the data than the uni-modal Wood's model at the 95% significance level. For this purpose, both models were separately fitted to the individual's log_10_ RT-PCR data and the Akaike's Information Criterion (AIC) was obtained. The AIC difference between the competing models for each individual pig is analogous to the likelihood ratio test statistic (D) when adjusted for twice the difference in the number of model parameters of the two models. The Wood's model has 3 parameters and the Extended Wood's model has 7 parameters, thus the AIC difference adjusted for the number of parameters becomes: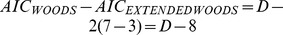. Thus at the 95% significance level the required likelihood ratio test, obtained from the chi-squared distribution, with 4 degrees of freedom, was 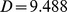 [36], corresponding to a critical difference in the AIC of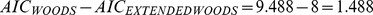. Thus, if the AIC difference was greater than or equal to this value (1.488) then the profile was classed into the biphasic rebound category, otherwise it was classed as uni-modal.

Transient experimental persistence henceforth referred to as persistence was defined as a subset within the non-rebound profiles according to the Wood's model prediction at the end of the experiment, i.e. at 42 dpi. If the model prediction at that time point remained above the detection threshold of one RT-PCR unit on the log_10_ scale then the profile was classified as ‘persistent’ within the 42 day observation period, otherwise it was referred to as ‘cleared’.

### Assessment of Individual Viremia Profile Properties

Description of the individual viremia profiles by analytical functions provides the opportunity to explore whether viremia features associated with different phases of the infection are related, and to construct and test hypotheses. Pearson product-moment correlations were calculated between individual Wood's and Extended Wood's function parameter estimates and the derived parameters -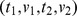 to determine whether general patterns were apparent in the fitted profiles (e.g. is there an association between viremia increase before the local peak and post-peak decrease, etc.). In particular, within the category of rebounders, associations between the shapes of the viremia curves describing the primary and the rebound phase of infection (i.e. 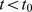 and 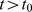, respectively) were tested.

Furthermore, to determine whether the phenomenon of transient persistence or viral rebound could be predicted by the shape of the profile during the earlier phase of infection, the Wood's model was fitted to the truncated dataset comprising observations from all pigs (rebounders and non-rebounders) from 0 to 21 dpi only. A linear mixed model analysis (using PROC MIXED of SAS 9.3) was then carried out to assess statistical differences in the individual Wood's curve parameter estimates associated with the different profile types (cleared, persistent and rebound), respectively. The dependent variables were the individual mean values of the estimated posterior distributions of the Wood's model and derived parameters obtained from the primary phase: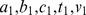. Fitted random effects were pen within trial and dam within trial. In the full model the fixed effects were the rebound class, trial, sex, parity and the interaction between trial and parity. Fixed and random effects were hierarchically removed according to statistical significance. Pairwise differences between the profile classes were assessed using the contrast statement of SAS PROC MIXED, which uses the F-test statistic.

### Association Between Viremia Profiles and Neutralising Antibody Response

To test the hypothesis whether viremia rebound, clearance or persistence was associated with greater within host viral diversity and thus more diverse neutralizing antibodies, logistic regression was carried out for the 439 pigs with nAB assays, using PROC GLIMMIX in SAS 9.3, assuming an exponential distribution of the data, conditional on random effects. In the full mixed model the dependent variable was the neutralising antibody class (i.e. binary as the nAB categories were pooled), the independent variables were the class of the viremia profiles (i.e. cleared, persistent or rebound). In addition, trial, profile class, parity, sex and corresponding interactions were fitted as fixed effects and dam within trial and pen within trial were fitted as random effects. Similar to the mixed model analysis above, the number of random and fixed effects in the full model was hierarchically reduced by examining their impact on the AIC model fit statistics, and according to statistical significance of individual fixed effects, respectively.

## Results

### Classification of Profiles

**Figure 2:**
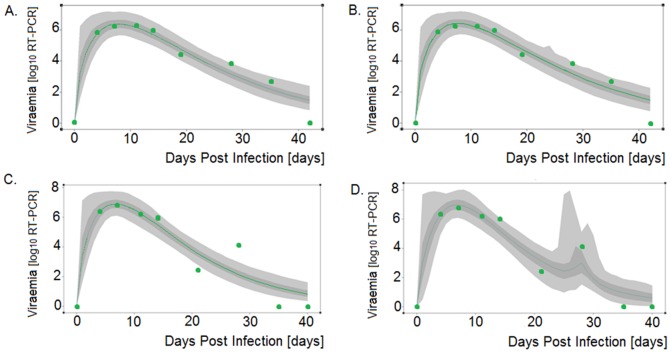
Wood's and Extended Wood's model fits to viremia data of two representative pigs classified as non-rebounder (top graphs) and rebounder (bottom graphs). Light-grey regions correspond to 95% posterior predictive intervals (PPI); dark-grey regions correspond to 50% PPIs. The actual data are shown as green dots and the green solid lines give the best-fit solutions for the Wood's model (A,C ) and Extended Wood's model (B, D), respectively.

Visual inspection of the predicted individual profiles (example shown in Figure 2) confirmed the appropriateness of the statistical classification method based on goodness of model fit. Only 12 and 4 of 1371 individuals had to be removed as outliers from the Wood's and Extended Wood's analysis, respectively, as several of their predicted viremia values differed from the corresponding observed values by more than 2 log differences. Overall 17% of the individuals were classified to experience viremia rebound while 83% were classified as non-rebounders (Table 1). Within the class of pigs with a uni-modal viremia profile, 46% of pigs were classified as pigs with persistent viremia whilst the remaining 54% of the non-rebounders appeared to have cleared the viremia. The percentages differed slightly between trials (Table 1). The lower percentage of rebounding profiles in trials 6–8 (7%, 6%, and 9% respectively), was possibly due to the higher number of missing values particularly in the later stage of the infection in these trials. Trails 7 and 8 were terminated at 35 dpi.

**Table 1:** Summary of the statistical classifications of the viremia profiles from the PRRSV challenge experiment

	Number (percentage) of individuals within a specific viremia profile category		
	Class 1: Uni-modal		Class 2: Biphasic
Trial	Clearance within 42 dpi	Persistence until 42 dpi	Rebound
1	79 (61)	50 (39)	55 (30)
2	119 (83 )	24 (17)	28 (16)
3	51 (37)	86 (63)	36 (21)
4	71 (47)	81 (53)	39 (20)
5	70 (48)	77 (52)	36 (20)
6	35 (35)	66 (65)	8 (7)
7	96 (55)	78 (45)	12 (6)
8	85 (59)	58 (41)	15 (9)
All (% of total)	606 (45)	520 (38)	229 (17)

% death prior to 21 dpi. Due to facility availability issues trials 7 and 8 had to be terminated at 35 dpi. Overall 17% were rebound, 38% were persistent and the remaining 45% were clearance profiles. Classifications of the viremia profiles are based on the likelihood ratio test comparing the Wood's and the Extended Wood's models. Trial 6 had 48

### Goodness of Model Fits

**Figure 3:**
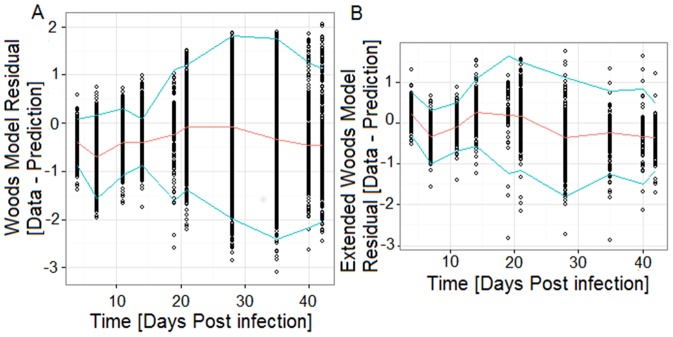
The Wood's model residuals (3A) and the Extended Wood's model residuals (3B). The red line shows the residual mean and the blue lines delimit two standard deviations from the mean.

**Figure 4:**
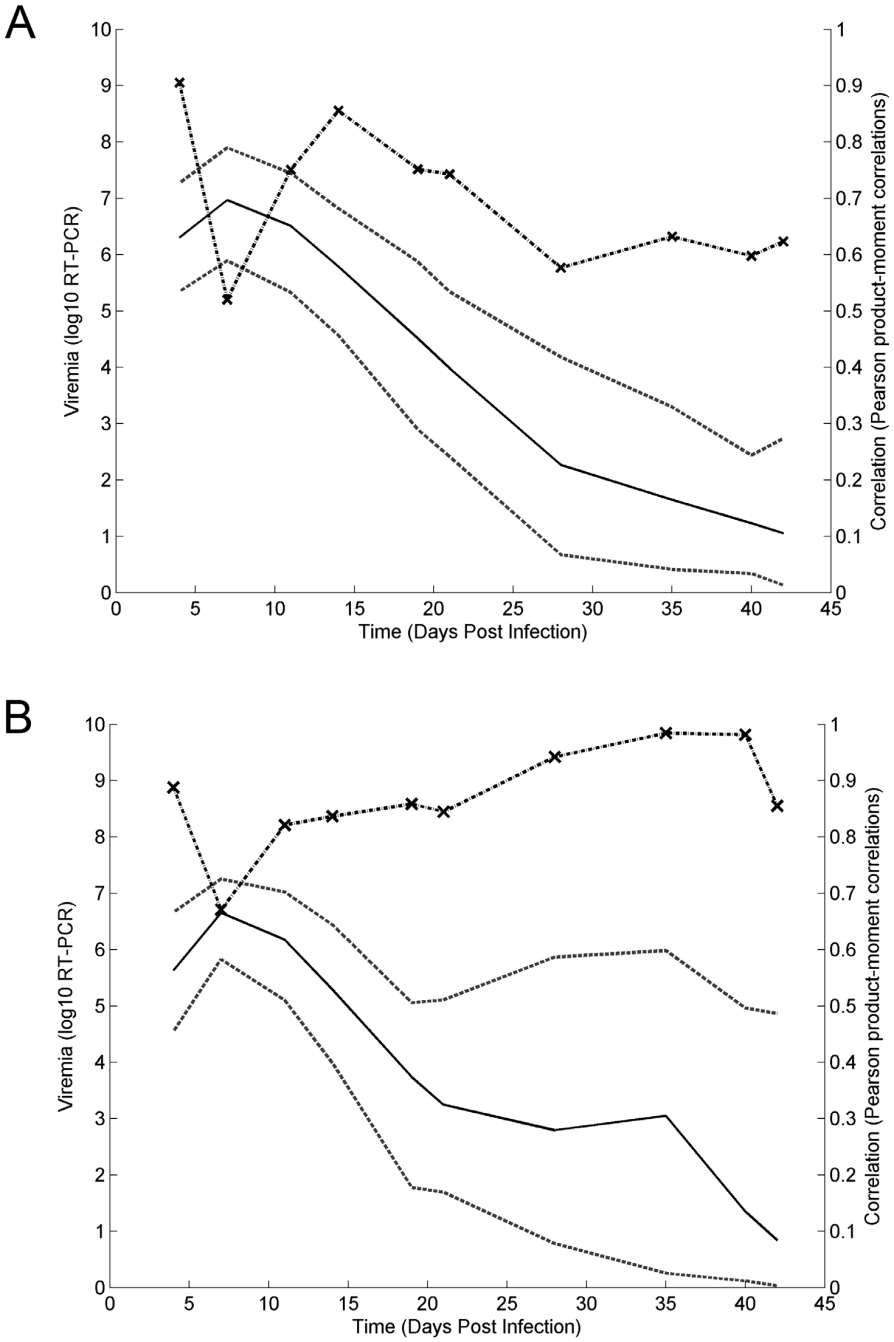
The Wood's(4A) and Extended Wood's(4B) model mean predictions and data-prediction correlations. The black lines outline the mean model predictions, and the dashed grey lines delimit the 95% prediction confidence intervals. The dotted black lines joined with crosses show the Pearson product-moment correlations between observed data and predicted values.

Inspection of the individual model fits (e.g. Figure 2), residual plots (Figure 3), and the Pearson product-moment correlations (Figure 4), revealed that the vast majority of profiles are adequately described by either the Wood's or the Extended Wood's model. The mean of the Wood's model residuals was close to zero at all sampling times and the majority of the residuals were within 2 standard deviations from the mean residual, with an increased residual variance and a slight tendency towards over-prediction from 28 dpi onwards (Figure 3). The Wood's model Pearson product-moment correlations (Figure 4) indicated that the model predictions and the data were highly correlated throughout the experiment. The average predicted time for the peak viremia was 7 dpi (Figure 4A), which coincided with the time of the second observation in the experiment, however a small subset of individuals with flat observations between 4 and 14 dpi contributed to a bias towards over-prediction at 7 dpi, leading to the lowest correlation between the predictions and the data being observed at this time point. The Wood's model also had a tendency to over-predict viremia at the late stages between 35–42 dpi, resulting from the fact that by model definition viremia levels are always positive (i.e. converge to zero but never reach zero), whereas data were truncated to zero when viremia was below the detection level. In fact, only 4 individuals had observations of viremia level below detection at 19 dpi and 21 dpi, however by 28 dpi 15% of the observations were below detection. By the end of the experiment 77% of the 492 individuals with viremia observations at 42 dpi were below detection.

The extended Wood's model gave a tighter plot of residuals (Figure 3B) with a reduced overall bias, and stronger correlations between observed data and fitted values (Figure 4B), than the Wood's curve. However, for individual pigs there were wide posterior predictive intervals (PPIs) around the second viremia peak for the biphasic profiles (see example in Figure 2D), with this uncertainty resulting from the fact that the viremic rebound was generally represented by only one or two data points. As with the Wood's model, the Extended Wood's model had a tendency to over-predict the initial peak and also to over-predict viremia around the second peak, a tendency most likely compounded by the sparse data around these points.

### Properties of Individual Viremia Profiles

#### Shape characteristics

The Pearson product-moment correlations between the individual model parameters (Table 2) reveal a strong relationship between the individual model parameter estimates describing the first mode of the viremia profiles 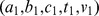, but not between the parameter estimates related to the second mode 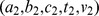, indicating a higher variation in the predicted individual profile shapes related to the rebound phase. In particular the estimates of the Wood's parameters 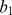 and 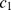 were highly correlated with a correlation of 0.92 and 0.87 for uni- and biphasic profiles respectively, indicating that a rapid increase to the peak viremia also corresponds to a rapid post peak viremia decline during this phase. This was confirmed by highly negative correlations (r = −0.84 and −0.88) between the derivatives of the viremia functions at 4 and 19/21 dpi for the Wood's and Extended Wood's model, respectively. This association did not occur for the rebound phase (the correlation between 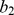 and 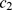 was 0.15). As expected, the time of the second peak 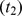 and the value for the time of onset of the rebound phase t_0_ were highly correlated; later onset of the secondary phase corresponded to a later time of peak viremia in the secondary phase. Correlations between the times and levels of peak viremia (i.e. between 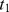 and 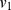, and between 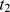 and 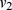, respectively ) were generally weak. Furthermore, the correlations between the corresponding Extended Wood's model parameters defining the first and second mode of the viremia curve, i.e. 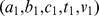 and 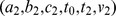, respectively, were generally weak, with the exception for a moderately strong negative correlation of −0.52 between the parameters 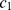 and 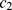. Thus a fast viremia decline in the primary phase tended to correspond to a slower decline in the secondary phase and vice versa. Interestingly, the analysis revealed an apparently strong negative association between the predicted peak viremia and subsequent decline associated with the rebound phase only (i.e. r(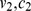) = −0.89, but r(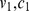) = 0.23).

**Table 2:** The Wood's and Extended Wood's model parameter's Pearson product-moment correlations.

Extended Wood's Parameters	a_1_	b_1_	c_1_	t_1_	v_1_	a_2_	b_2_	c_2_	t_0_	t_2_
a_1_	-	**−0.91**	**−0.71**	**−0.81**	**0.04**					
b_1_	−0.85	-	**0.92**	**0.59**	**0.21**					
c_1_	−0.52	0.87	-	**0.26**	**0.26**					
t_1_	−0.84	0.52	0.39	-	**0.004**					
v_1_	0.19	0.12	0.23	−0.16	-					
a_2_	−0.05	0.07	0.10	−0.02	−0.01	-				
b_2_	0.02	0.01	0.04	−0.03	0.02	−0.10	-			
c_2_	0.03	−0.30	−0.52	0.33	−0.27	−0.08	0.15	-		
t_0_	0.15	−0.14	0.07	−0.13	0.05	−0.04	−0.09	−0.09	-	
t_2_	0.17	−0.10	0.57	−0.20	0.10	−0.06	−0.05	−0.24	0.98	-
v_2_	0.07	0.25	0.47	−0.33	0.30	0.14	0.08	−0.89	0.13	0.29

Upper triangle in bold: Wood's model parameter correlations. Lower triangle: correlations between the Extended Wood's model parameters.

#### Is viremia clearance, persistence or rebound predictable

**Figure 5:**
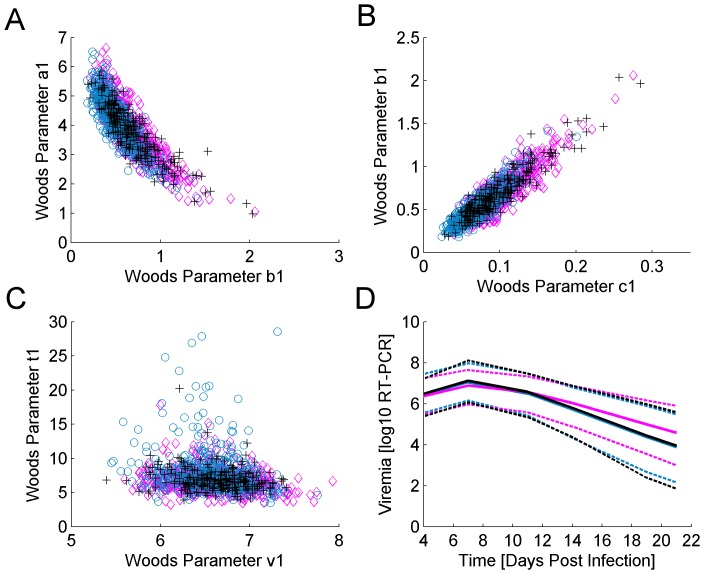
Woods parameters and mean model predictions for the 3 profile classes. Figure 5A-C. Pair-wise scatter plots and clustering of primary phase Wood's model parameters for the 3 profile classes: cleared (blue circles), persistent (pink diamonds) and rebound (black crosses). Figure 5D. Mean Woods model predictions with the 95% Confidence Intervals (dashed lines) based on the truncated data from 0–21 dpi for individuals previously classified as rebound (black), persistent (pink) and cleared(blue), respectively.

The final mixed models for original and derived Wood's model parameters obtained from the truncated data from the primary phase only (0–21 dpi), included fixed effects of profile class (cleared, persistent or rebound) determined on the complete dataset, as well as the trial and the trial by parity interaction, with pen within trial and dam within trial as random effects. Statistically significant differences for all the individual Wood's curve parameter estimates (i.e. 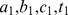 and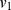 ) were found between animals classified into the cleared and persistent viremia classes, and between those classified as persistent and rebounders, but not between animals classed as cleared and rebound (Table 3). Inspection of the pairwise scatter plots of Wood's model parameter estimates (Figures 5A-C) shows clustering associated with the different profile categories, in concordance with the statistical test statistics (Table 3). Furthermore, identified differences between parameters corresponding to persistent and non-persistent profiles are also transparent in plots of the mean viremia predictions together with their 95% confidence intervals (Figure 5D). Thus, the results suggest that whereas persistence is predictable based on the profile of the first 21 dpi, viremia rebound is not.

**Table 3:** Viremia profile class and their Least Square Means (LSM) from the truncated data.

Wood's parameter	LSM (SE): Cleared	LSM (SE): Persistent	LSM (SE): Rebound	P_CP_	P_CR_	P_PR_
a_1_	3.83 (0.049)	4.16 (0.051)	3.84 (0.066)	<.01	0.98	<.01
b_1_	0.68 (0.011)	0.53 (0.012)	0.68 (0.017)	<.01	0.91	<.01
c_1_	0.097 (0.0014)	0.072 (0.0015)	0.096 (0.0022)	<.01	0.92	<.01
t_1_	6.90 (0.11)	7.82 (0.12)	6.99 (0.16)	<.01	0.58	<.01
v_1_	6.60(0.018)	6.56 (0.019)	6.61 (0.024)	0.02	0.78	0.04

–21 dpi for pigs whose viremia profiles were classified as cleared, persistent and rebound, respectively. P-values corresponding to the associated test between the profile groups denoted by the subscript C, P, R for cleared, persistent and rebound respectively. The LSM parameters estimates and standard errors (SE) derived from fitting the Wood's model to the truncated dataset from 0

### Association Between Viremia Profiles and Neutralising Antibody (nAb) Response

The percentages of individuals in each nAb category for each viremia profile class are presented in Table 4 and odds ratios of nAb cross-productivity associated with different viremia profiles classes are presented in Table 5. The final model used to test the association between the profile-class and the nAb response contained only the nAb class as dependent variable and the viremia profile class as independent variable, with no other fixed or random effects. There was a statistically significant association (at the 95% significance level) between the profile class and the diversity of the nAb response when the nAb categories were pooled into two groups (i.e. no nAb cross-protection and cross-protection). Clearance corresponded to a less diverse nAb response than persistence and pigs with persistent viremia had a more diverse nAb response than those with rebound profiles (Table 5). However, there was no statistical significant difference in nAb cross-protectivity between pigs that cleared viremia within 42 dpi and rebounders (Table 5).

**Table 4:** Neutralising antibody (nAb) data and viremia class.

nAb category (1–5)	Class	Number Of Individuals	Percentage [% of Class]
1	Rebound	6	5.9
2	Rebound	55	53.9
3	Rebound	11	10.8
4	Rebound	21	20.6
5	Rebound	9	8.8
Total (1–5)	Rebound	102	-
1	Cleared	21	9.4
2	Cleared	104	46.6
3	Cleared	51	22.9
4	Cleared	33	14.8
5	Cleared	14	6.3
Total (1–5)	Cleared	223	-
1	Persistent	16	14
2	Persistent	61	54
3	Persistent	21	18
4	Persistent	11	10
5	Persistent	5	4
Total (1–5)	Persistent	114	-

–5 correspond to nAB response to the homologous strain and k-2 other PRRSV strains. Frequency and percentages of individuals classified as cleared, persistent and rebound, within each neutralizing antibody (nAB) category. nAB category 2 refers to individuals whose serum contains nAB that can only neutralize the homologous PRRSV strain (NVSL) as used in the in-vivo infections; nAB categories 3

**Table 5:** Odds ratios of the cross protectivity of neutralising antibodies (nAbs) from individuals from profile Class 1 relative to that of nAbs from individuals from viremia profile Class 2.

Class 1	Class 2	Odds ratio (95% Confidence interval)	P- value
Clearance	Persistence	0.61 (0.38, 0.98)	0.04
Clearance	Rebound	0.86 (0.53, 1.38)	0.53
Persistence	Rebound	1.40 (0.80, 2.45)	0.24

–2) or not cross-protective (nAb class 3–5). The 95% significance level was used (p<0.05). For further explanation see text. The columns class 1 and class 2 indicate which two viremia categories are being compared. The nAb class was a pooled into a binary trait: cross-protective (nAb class 1

The results thus suggest that the slower clearance rate of viremia in the persistent profiles, but not viremia rebound, is associated with a more diverse nAb response.

## Discussion

Empirical mathematical models have proven useful for describing the temporal evolution of a response variable and filtering stochastic noise from dynamic biological systems whilst retaining the most fundamental features [31], [37]–[39]. Previous studies have used such models for the quantitative analysis of growth, weight gain and lactation [37], [40]; however to our knowledge this is the first study in which they have been applied to infection profiles. The comprehensive PHGC dataset comprising repeated PRRS viremia measurements from 1371 commercial pigs provided a unique opportunity to assess the nature and degree of variation in the host response to PRRSV infection. The main aims of this study were to obtain a mathematical function to aid the characterisation and to further our current understanding surrounding the wide spectrum of observed PRRS viremia profiles.

Using maximum likelihood inference and numerous model fit criteria, we established that the broad spectrum of observed viremia trends from 0–42 dpi could be adequately represented by either the uni- or biphasic Wood's functions. Representative parameter estimates of the data resulting in good model fits and residuals were obtained for the vast majority of individuals, and pigs could be objectively classified into one of three viremic categories. This together with the convenient biological interpretation of the model parameters and derived parameter estimates, such as the time and level of viremia peak, allowed us not only to quantify inter-host variation, but also to establish common viremia curve characteristics and determine their predictability.

Assessment of the fitted models revealed a generally close fit of the Wood's and Extended Wood's model to the log transformed viremia data over the whole 42 day duration of the experiment, with few exceptions. The lowest data-prediction correlation was observed at 7 dpi, which corresponds to the average time for the peak viremia level. The residuals showed that for certain individuals the model over-predicts the value at this observation. There are two main factors which may contribute to this lack of fit: firstly for individuals whose peak viremia may lie between 4–7 dpi, the lack of data representing the dynamics of the infection during this period of rapid change may contribute to the over-prediction. Secondly for individuals with a fairly flat plateau of observations between 4–14 dpi the issue lies with the Wood's model itself; in this case the model systematically predicts a higher and sharper peak than the data would suggest as it is unable to produce a flat plateau near the peak. The Wood's (and Extended Wood's) model itself is constrained by the model parameters; the time of the peak viremia level is dependent on both parameters 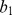 and 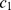 which in turn contribute to the rates of pre-peak viremia increase and post-peak viremia decline. In contrast, the general tendency to over-predict viremia from 28 dpi onwards for non-rebounders can easily be explained by the fact that the Wood's curve converges to, but never reaches, zero. This latter property of the Wood's function may in fact be a clearer representation of reality; we are never able to confirm that viremia levels truly reach zero, but only that they have reached levels below the detection threshold determined by the accuracy of experimental observations. Note that theoretically the log- transformed viremia data could reach negative values which the Wood's model would be unable to capture. However such viremia observations never arise in practice due to the experimental threshold of detection and hence a function such as the Wood's model that approaches zero is appropriate for the current PRRS viremia dataset.

By parsimony, in the absence of more frequent measurements, the Extended Wood's model was chosen as it is a simple model representing the main features of the biphasic profiles. It encapsulates the assumption that the second phase of the profile has the same essential shape characteristics as the primary phase. Furthermore the Extended Wood's model parameters were also able to encapsulate the possible anamnestic nature of the immune response; the parameters allowed for variation in the size, timing, rate of increase and rate of decline in the secondary phase. The consistency of the model residuals and correlations indicate that the Extended Wood's model provides indeed a good fit during both phases of the profile; in fact the correlations between the data and model predictions were highest during the second phase. The second phase was generally shorter (28–42 dpi) and less severe than the first phase (0–28 dpi), as indicated by greater predicted rates of viremia increase and decline in the second phase of the profile (parameter means for the primary and secondary phase were: mean(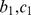)  = (0.66,0.10) and mean(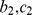)  = (4.67,3.78)), and lower levels of peak viremia associated with the second phase. This contrast in timing and size of the primary and secondary phases supports evidence of the anamnestic nature of an individual's immune response in a rebound profile.

Despite the generally good fit of the Wood's and Extended Wood's models, it is likely that a candidate model of greater mathematical complexity could provide a better empirical fit to the data; indeed much effort has been dedicated to the identification of appropriate functions to describe e.g. lactation or growth profiles in livestock [31], [40]. Amongst these candidates are spline functions, which indeed were able to provide a closer fit to the present viremia data (results not shown). However their increased complexity requires biological interpretation and does not lend itself to a quantitative assessment of profile characteristics. The Wood's and Extended Wood's models in contrast constitute worthy candidates for this analysis, particularly as biologically meaningful interpretations can be given to their parameters.

The 42 day study period gave rise to uni- or biphasic viremia profiles only. It is possible that the observed biphasic profiles represent damped oscillations which are truncated after 6 weeks post infection. Oscillations often represent negative feedbacks with delay in biological systems [41]. Such behaviour could arise from the virus-host immune response interactions. An oscillatory mathematical function such as a sine function may constitute a plausible alternative to the Wood's functions presented here and would be attractive from the mathematical perspective due to the few parameters needed to determine the period of the cycles and damping factor of the oscillations. However the current dataset doesn't span a sufficient duration to inform parameter estimates for such models; data from longer PRRS virus challenge experiments would be required to test these hypotheses.

One of the advantages of fitting alternative mathematical functions to viremia data is that it provided an objective method for distinguishing uni-modal from biphasic viremia profiles based on statistical inference. This helped to confirm previous observations from smaller scale studies (e.g. [17] and [20] ) that viremia rebound is a genuine and common phenomenon in PRRSV infections. This may have important epidemiological consequences, as individuals diagnosed as non-viremic at time of sampling may harbour and shed infectious virus some days later. Similarly, individuals with persistent viremia profiles are likely to be infectious for longer. The question thus arises whether serum viremia persistence or rebound can be predicted, e.g. based early profile characteristics. Our statistical analyses, using data from the primary phase (0–21 dpi) of the experimental infection, revealed that profiles classed as persistent had, on average, a faster increase to the peak and slower decline from the peak viral load than both the rebound and clearance profiles. However, the results also suggest that rebound and clearance profiles cannot be distinguished based on information from the primary phase. This result has however important implications with regards to the following hypothesis: considering that serum viremia data were only collected for 42 dpi, one may hypothesise that every pig may eventually experience rebound, provided that the virus has not been completely cleared, and that rebound could only be observed for a subset of pigs in this study due to censoring. However, if this hypothesis was correct, there would be a higher probability of observing rebound in individuals with faster viremia decline within 21 dpi. But, since no statistical difference was observed between rebound and clearance on the truncated dataset, the existing evidence would suggest that only a subset of pigs experiences viremia rebound. Furthermore, we also observed that none of the pigs with non-detectable viremia levels for 2 or more weeks experienced viremia rebound within 42 dpi (results not shown), thus indicating that rebound is unlikely to occur if the serum virus has been cleared for a period of several weeks.

Analysis of the nAbs of infected pigs collected at 42 dpi indicated that there was a strong homologous response to the PRRSV challenge, in agreement with observations from previous studies [42], [43]. Our study not only revealed a high degree of inter-pig variation in viremia profiles but also high variability in the range of cross-protection of neutralizing antibodies collected at 42 dpi. Furthermore, a statistical association between the two quantities was identified, which may point to the host-pathogen interactions underlying the observed viremia and antibody patterns. Both virus persistence within the host and cross-protection of host antibodies have been previously linked to virus mutation and emergence of quasi-species [9], [38]. PRRSV evolves rapidly in infected pigs with a high mutation rate of 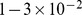 substitutions per nucleotide and year [21], [44]. Furthermore, multiple variants of PRRSV were found to exist simultaneously within individual animals [22]. Thus, in order to efficiently clear the range of PRRSV quasi-species, a versatile neutralizing antibody response would be required.

Our study revealed that pigs that manage to clear the virus from the blood within 42 dpi have on average a less diverse nAB response than pigs with persistent viremia profiles. In light of the pathogen co-evolution, these results could be interpreted as rapid clearance of the virus limiting the duration in which the virus may evolve, resulting in an efficient, but relatively narrow antibody response. Persistent profiles, in contrast, may be the result of a host immune response that is inefficient in clearing the virus but in which the diversification of the neutralizing antibody response is driven by rapid virus mutation. Rebound is characterised by two phases; the viremia decline in the first phase may be caused by an effective, but narrow nAb response. However, before the immune response has managed to fully clear the virus within the primary phase, the emergence of a new quasi-species capable of escaping the existing antibodies may cause the viremia to increase in a second phase driving the continual diversification of nAbs that eventually reduce viremia again. Thus, given that the majority of rebound profiles are at the declining phase by 42 dpi, viremia rebound would be expected to be associated with a more versatile nAb response at 42 dpi than clearance, but a more narrow response than persistence, which is in agreement with the results, although the differences were not always found to be statistically significant.

It should be noted, however, that the observed associations between the nAB response and viremia patterns may equally arise from confounding immune quantities affecting both nAB and viremia, which were not measured in these studies. Indeed, several alternative hypotheses, not necessarily involving virus mutation, emerge as potential causes for viremia rebound. For example viremia rebound may correspond to the second cycle of an oscillating viremia profile which may arise naturally (and in the absence of virus mutation) from predator-prey type interactions between the virus and the immune response [45], [46]. Alternatively, rebound could arise from the heterogeneity of the virus distribution in various tissues within the host. Previous studies have observed that although viremia may be below detection in the serum, persistent infection in the tonsils and lymphoid tissues can last for longer than 6 months [18]. Thus, the site of replication may be hidden from the serum and the infection may remain localised in certain tissues. Rebound may be a manifestation of the virus from these localised tissue infections being poured back into the system; thus becoming detectable in the serum. The influx of virus into the serum may occur in occasional bursts or as a final out-pouring into the serum determined by some environmental stimulus, immune response mechanism, or stochastic process. This second hypothesis would imply that viremia rebound follows more a distinct bi- or multi-modal pattern rather than a damped oscillatory behaviour. Virus rebound manifesting itself in biphasic viremia profiles is a common phenomenon in equine influenza infections, the causative processes of which remain a mystery [47]. Additional information about virus heterogeneity and/or nAb characteristics at different stages post infection would be needed to assess these hypotheses.

Similar arguments may be used to interpret the observed high inter-host variation in viremia decline post the initial viremia peak. Variation in the rate of viremia reduction may be a function of the host's own immune response, virus mutation mechanisms or the interaction between both processes. The moderate negative correlation between parameters 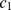 and 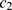 (-0.52) indicates that a slow decline in the first cycle corresponds to faster decline in the second cycle and vice versa. This association may be indicative of the temporal evolution of the immune response. A slow immune response in the primary phase may prolong the conditions required to produce neutralising antibodies and thus manifest in a stronger or more diverse nAb at the end of the second phase. Conversely, a fast initial immune response may indicate an effective, but narrow nAb response during the primary phase so that fewer or less diverse nAB are available for the second phase. The identified association between nAb and viremia categories would support this hypothesis, since persistent profiles have the broadest response of all the profile categories.

From the current study we cannot affirm that there would be infectious and epidemiological consequences in the secondary phase of rebound profiles. Inferences made on infectiousness and epidemiological consequences using the data obtained via quantitative RT-PCR may be limited due to the potential discrepancy between the measured viral genome load and the non-measured true viral load. Thus, the observed secondary phase of rebound may be the result of detecting circulating junk genomes rather than genomes of infectious particles and may thus have no significant epidemiological consequence. However, previous studies have explored the relationship between diverse viremia measurements an infectiousness of pigs infected with PRRSV [29], [48], [49]. For example, Charpin et al. [48] detected viral genome in the blood of inoculated pigs from 7–77 dpi using RT-PCR, whereas viral genome shedding was detectable from nasal swabs from 2–48 dpi. Furthermore their study concluded that infectiousness was indeed correlated with the time course of viral-genome in the blood measured by RT-PCR and that the decrease in infectiousness was related to the increase in antibodies[48]. In a study by Rowland et al.[49]it was observed that even when there were low levels of the virus replication the virus was easily transmitted and it was only by 260 dpi that pigs were no longer able to transmit the virus to sentinel pigs[49]. Thus it has been established that some PRRSV-infected pigs can support virus replication and transmit infection for an extended period far beyond the duration of the secondary phase of the profiles in our study. By this logic one may argue that the observed rebound in viral genome load may indeed have biological consequences in terms of viral shedding and transmission to susceptible animals.

Previous PRRS studies alluded to the phenomenon of viremia rebound [14], [20], [50] based on qualitative inspection of viremia profiles, but have not analysed the duration, defining characteristics or relationship with the antibody response of this phenomenon. Using data from the first 3 out of the 8 trials analysed in this study, Boddicker et al. [20] defined biphasic profiles subjectively by a rebound of 2 units on the logarithmic (base 10) scale of viremia observations from 21 dpi onwards. Using this definition rebound was not found to be heritable, indicating that rebound is more likely to be determined by either the virus or the environment as opposed to host genetics [20]. However, the low heritability estimate of this phenotypic trait (0.03) may be due to poor trait definition arising from too few successive observations to accurately capture the rebound phase (i.e. between 21–42 dpi), and hence errors in classifying animals. The statistical classification used in this study resulted in 22% of the pigs from trials 1–3 classed as rebounders as opposed to the 33% of pigs classed into the rebound category in the previous study [20].

Lastly, there is accumulating evidence for substantial host genetic variation in response to PRRSV [1], [4], increasing the potential for the control of PRRS through genetic selection. In particular, a quantitative trait locus (QTL) was identified for the area under the viremia curve spanning the first 21 days post infection [20]. Our study provides opportunity to assess whether clearance, persistence or rebound, and other derived features, such as level of or time to peak viremia and the rates of increase and decline in the profiles are genetically determined, and to potentially identify QTL associated with these new phenotypes. The new derived phenotypes may provide deeper insights into the underlying molecular mechanisms. Furthermore, the hypotheses for host-pathogen interactions emerging from the current study will be used to inform and validate process-based dynamic mathematical models of *in-vivo* PRRSV infections in future studies.
